# Disinvestment in the presence of uncertainty: Description of a novel, multi-group, disinvestment trial design and protocol for an application to reduce or cease use of mobilisation alarms for preventing falls in hospitals

**DOI:** 10.1371/journal.pone.0261793

**Published:** 2021-12-30

**Authors:** Terry P. Haines, Mari Botti, Natasha Brusco, Lisa O’Brien, Bernice Redley, Kelly-Ann Bowles, Alison Hutchinson, Debra Mitchell, Joanna Jellett, Kate Steen, Leanne Boyd, Melinda Webb-St Mart, Melissa Raymond, Peter Hunter, Phillip Russo, Rachel Bonnici, Dai Pu, Samantha Sevenhuysen, Vicki Davies, Ronald Shorr

**Affiliations:** 1 School of Primary and Allied Health Care & National Centre for Healthy Ageing, Monash University, Frankston, Australia; 2 School of Nursing & Midwifery, Deakin University, Geelong, Australia; 3 Rehabilitation, Ageing and Independent Living (RAIL) Research Centre, School of Primary and Allied Health Care, Monash University, Melbourne, Australia; 4 Department of Occupational Therapy, Monash University, Melbourne, Australia; 5 Centre for Quality and Patient Safety Research-Monash Health Partnership, Melbourne, Australia; 6 School of Nursing & Midwifery, Faculty of Health, Deakin University, Geelong, Australia; 7 Department of Paramedicine, Monash University, Melbourne, Australia; 8 Allied Health Workforce, Innovation, Strategy, Education and Research (WISER) Unit, Monash Health, Clayton, Australia; 9 Falls Prevention Service, The Mornington Centre, Peninsula Health, Victoria, Australia; 10 Epworth HealthCare, Richmond, Australia; 11 Chief Nursing and Midwifery Officer, Executive Director Learning and Teaching, Eastern Health, Richmond, Australia; 12 Eastern Health, Richmond, Australia; 13 Physiotherapy Department, Alfred Health, Melbourne, Australia; 14 College of Science, Health and Engineering, La Trobe University, Melbourne, Australia; 15 Geriatric Medicine, Alfred Health, Melbourne, Australia; 16 School of Nursing & Midwifery, Monash University, Melbourne, Australia; 17 Department of Nursing Research, Cabrini Institute, Malvern, Australia; 18 Peninsula Health, Frankston, Australia; 19 Subacute Ambulatory Care Manager Peninsula Health, Frankston, Australia; 20 Geriatric Research Education and Clinical Center, Malcolm Randall Veterans Affairs Medical Center, Gainesville, Florida; 21 Department of Epidemiology, University of Florida, Gainesville, Florida; Public Library of Science, UNITED KINGDOM

## Abstract

Disinvestment is the removal or reduction of previously provided practices or services, and has typically been undertaken where a practice or service has been clearly shown to be ineffective, inefficient and/or harmful. However, practices and services that have uncertain evidence of effectiveness, efficiency and safety can also be considered as candidates for disinvestment. Disinvestment from these practices and services is risky as they may yet prove to be beneficial if further evidence becomes available. A novel research approach has previously been described for this situation, allowing disinvestment to take place while simultaneously generating evidence previously missing from consideration. In this paper, we describe how this approach can be expanded to situations where three or more conditions are of relevance, and describe the protocol for a trial examining the reduction and elimination of use of mobilisation alarms on hospital wards to prevent patient falls. Our approach utilises a 3-group, concurrent, non-inferiority, stepped wedge, randomised design with an embedded parallel, cluster randomised design. Eighteen hospital wards with high rates of alarm use (≥3%) will be paired within their health service and randomly allocated to a calendar month when they will transition to a “Reduced” (<3%) or “Eliminated” (0%) mobilisation alarm condition. Dynamic randomisation will be used to determine which ward in each pair will be allocated to either the reduced or eliminated condition to promote equivalence between wards for the embedded parallel, cluster randomised component of the design. A project governance committee will set non-inferiority margins. The primary outcome will be rates of falls. Secondary clinical, process, safety, and economic outcomes will be collected and a concurrent economic evaluation undertaken.

## Introduction

Improving patient outcomes and the efficiency of service delivery requires existing techniques, therapies, technologies, and service delivery models to change over time. The term “disinvestment” has been referred to as the displacement of non-cost-effective approaches for resource reinvestment or reallocation [1]. Disinvestment can be applied equally to approaches that are clearly ineffective or harmful, as to interventions that were once shown to be beneficial and cost-effective but are no longer when compared to new, competing approaches (obsolescence). Similarly, it can apply when an intervention is used more often than is indicated (over-use), or for purposes other than those for which it was originally intended in the absence of evidence that doing so is clinically effective and cost-effective (misuse) [1].

One of the five principal challenges in disinvestment is the lack of published studies which clearly demonstrate that existing technologies/practices provide little or no benefit [2]. This not only makes classification of a practice as clearly being low-value more difficult, it may serve to deprioritise that area to be the subject of a disinvestment process [3, 4], and make the disinvestment process less acceptable to stakeholders, potentially leading to resistance and failure to sustain the disinvestment [5, 6].

Several authors have argued that disinvestment efforts should be focused upon areas with strong evidence of ineffectiveness (also inefficiency or harm) [3, 4]. We agree that disinvestment should take place when this is the case, and propose that disinvestment efforts may also be directed to areas of practice where evidence of benefit is missing yet we cannot be certain that they are ineffective. The amount of health care resource being consumed for practices with uncertain effect is likely to be large. There is documented evidence of overuse or misuse of numerous health technologies and those that simply have no high-quality evidence of effectiveness in any context [7, 8]. Generating more evidence of the effectiveness of these practices is likely to confirm that a number are ineffective. A 2013 review of 2044 articles published in major general medical journals that examined an established practice found 40% reported evidence for practice reversal [9]. Previous authors have argued that the rate of development of a stronger evidence base in areas of routine practice is likely to be slow given the difficulties with generating evidence in these contexts [3, 10]. The likely outcome of this is that routine practices of questionable benefit will continue, new evidence to support ineffectiveness will only slowly be generated, and resources will continue to (potentially) be wasted.

### Disinvestment while simultaneously generating evidence

A new approach to managing disinvestment from routinely provided health services where there is some, but insufficient evidence of ineffectiveness has previously been described [10]. This strategy has been to disinvest from a routinely provided health practice while simultaneously generating evidence required that would render an assessment of ineffectiveness much more certain. This approach involves adaptation of a conventional stepped-wedge, cluster randomised trial design conducted under a superiority paradigm to become a disinvestment-specific stepped-wedge conducted under a non-inferiority paradigm.

**Fig 1:**
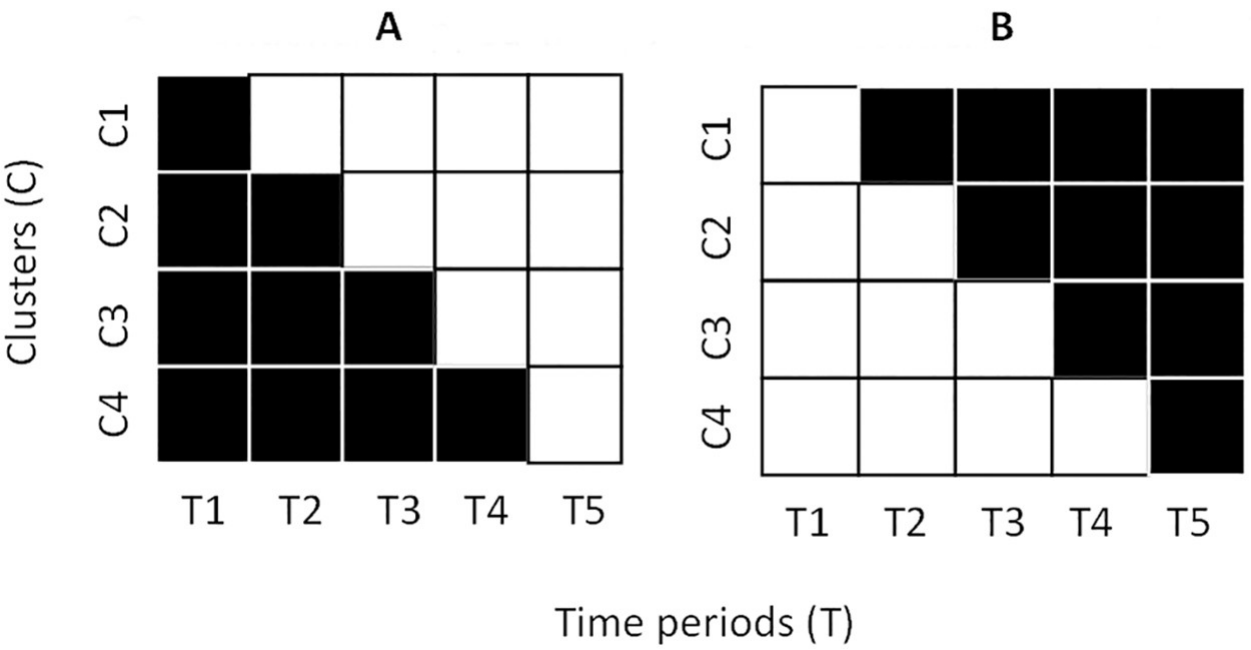
A. Conventional stepped-wedge. B. Disinvestment stepped-wedge. Black = control period. White = intervention period.

A conventional stepped-wedge cluster randomised trial [11] is a unidirectional cross-over design (Fig 1A). It involves the roll-out of an intervention in a randomly determined order across a number of clusters such that each cluster is exposed to both control and intervention conditions. This also allows for maturation effects (subjects and clusters changing naturally over time) to be accounted for through statistical adjustment of time period within the analysis approach [11]. A disinvestment stepped-wedge reverses the order of the conventional roll-out approach, such that each cluster commences with the routinely provided candidate approach in place and then has it removed in a randomly determined order (Fig 1B) [10]. Non-inferiority margins and stopping rules are established though stakeholder engagement processes, and data are periodically examined to monitor safety and unforeseen consequences. The key advantages of this approach have been argued as being that:

1. The stepped-wedge (as compared to a parallel cluster design) minimises the number of patients exposed to the condition where the candidate approach being disinvested from has been withdrawn from the outset of the trial.
2. Data can be examined incrementally for harms and stopping rules implemented rapidly by an independent data monitoring committee to see whether changing the routinely provided intervention has unanticipated consequences.
3. Stakeholders, who are likely to be anxious about and a key barrier to the disinvestment, can have a meaningful role in trial design, particularly through contributing to outcome measure selection, and helping to set non-inferiority margins and stopping rules.
4. Generation of robust data through the disinvestment trial has been found to be a key enabler for gaining acceptance for undertaking this disinvestment strategy [6].

### Multiple levels of intervention, and limitations of the sequential approach

The first application of this approach examined whether disinvesting from weekend allied health services delivered to acute medical or surgical wards in hospitals would have a detrimental effect on patient and health service outcomes [12]. Stakeholder consultations during project planning revealed that more than one “intervention” condition that was considered relevant by our stakeholders. The initial plan for this project was to compare the weekend allied health services that were currently in place with a “no weekend allied health service condition”. However, feedback received indicated that if the “no weekend allied health service condition” was found to be non-inferior to the “current” weekend allied health service condition, then clinicians at other hospitals would ignore the results, using the justification that the “current” weekend allied health service model at these hospitals was poorly constructed in the first place and their own models were different and better. This highlighted the need to encompass multiple intervention models within the same disinvestment study.

**Fig 2:**
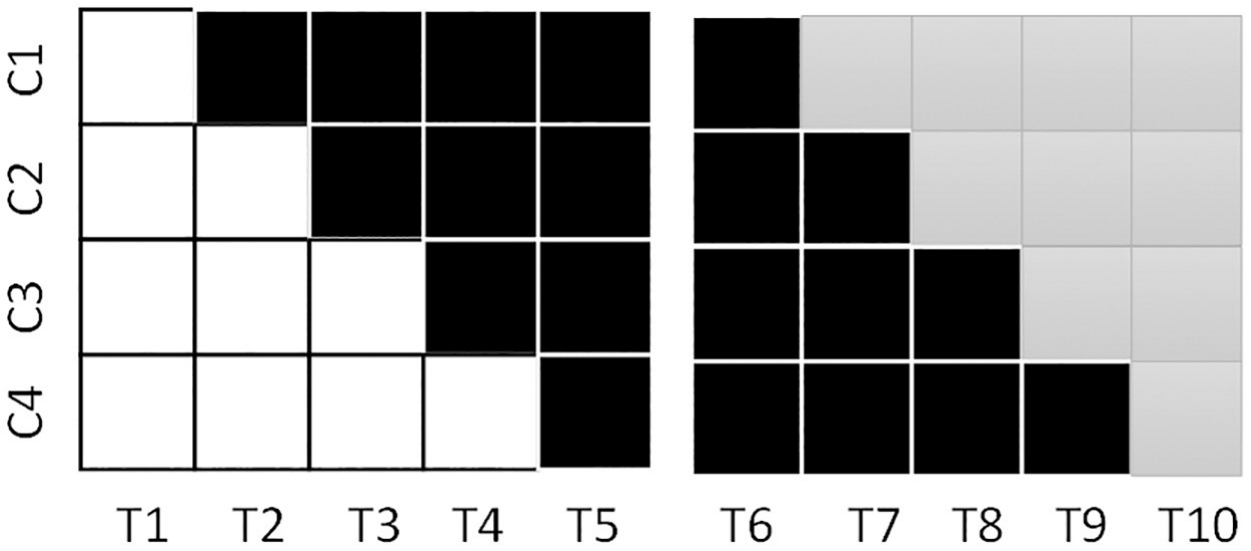
Disinvestment stepped-wedge followed by conventional. White = current service condition, Black = no service condition, Grey = newly developed service condition.

To respond to this feedback, a sequential trial approach was used, where the planned disinvestment trial (current service vs no service models) was followed by a conventional stepped-wedge trial where a newly developed service (developed through stakeholder consultation, use of data and feedback from the first trial, and a Delphi decision-making process among allied health managers at each site) within the same budget envelope was re-introduced on the same wards. An abbreviated conceptualisation of this is presented in Fig 2.

Three limitations of this sequential 3-group trial solution were identified. First, the trial ends in a condition of providing the newly developed weekend allied health service. If this service was to be found to be ineffective, an additional process of disinvestment would be required, creating an additional barrier to implementing the disinvestment. Second, the sequential trial approach could only test two of the three possible pairwise comparisons directly. Comparison of the two intervention conditions is contaminated by maturation effects as they did not have overlapping time periods. This was borne out in the results to some extent as the no-service conditions in each trial were different across multiple outcomes when they were compared [12]. Third, this approach takes a long period of time, within which service changes can create unwanted complications in the design. In that study, one of the wards was closed by the hospital, reducing the number of wards in the second trial.

### A new approach for multiple-group, disinvestment comparisons

**Fig 3:**
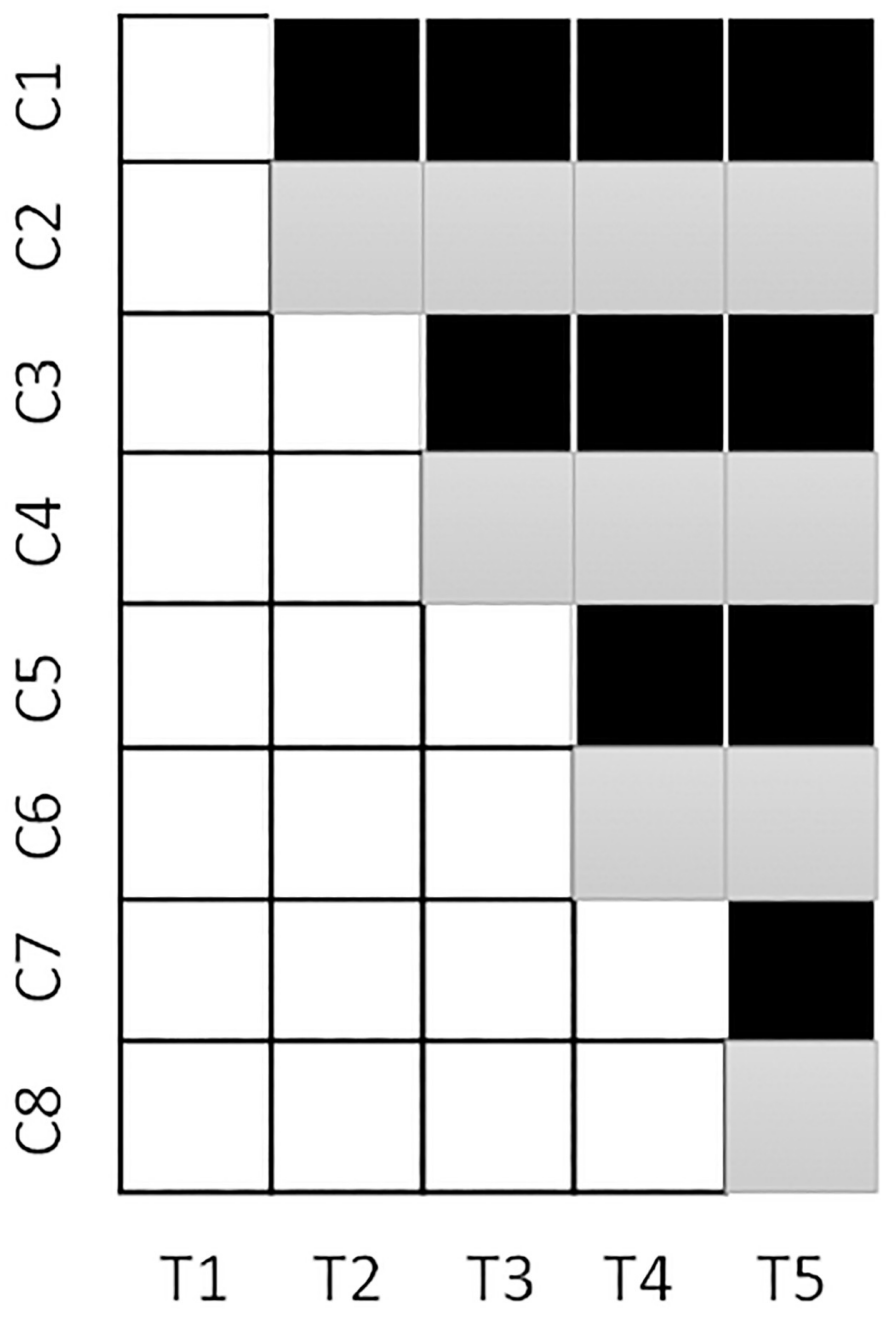
3-group, concurrent, disinvestment trial. White = current service condition, Black = no service condition, Grey = alternate service condition.

Our new solution is to employ a multiple-group, concurrent, disinvestment, stepped-wedge with an embedded, parallel, matched-cluster, randomised trial design (Fig 3 displays a 3-group configuration). In essence, this design merges multiple disinvestment trials concurrently. From Fig 3, if this approach had been applied to the weekend allied health service trial, there would have been a stepped-wedge comparing the current service to a no service condition and a stepped-wedge comparing the current service condition to an alternate service condition. Their concurrent combination creates a parallel, cluster randomised trial comparing the no service condition to the alternate service condition. This latter component of the trial could have been conducted through a superiority research paradigm. The design requires two stages of randomisation, the first to allocate each cluster to a particular time period for transitioning from the “current” condition, the second to split each cluster pair to either of the no service or alternate service conditions. Separating the randomisation in this way allows for dynamic randomisation to be used at the second randomisation point to promote balance between the no service and alternate service conditions based on the trial primary outcome(s) as they enter this period. Initial matching of clusters that transition at the same time point (e.g. matching C1 & C2) will also promote balance in this respect. Conducting a multiple-group comparison in this way generates improved statistical power across the comparisons [13], and ensures there is sufficient overlap in time period exposure for each comparison of interest (unlike the three-group sequential approach used previously).

## Application and trial protocol: Background

Mobilisation alarms are a staple in hospitals as part of the falls prevention management repertoire; they can take up to 11% of all falls prevention management costs [14]. However, a 2018 Cochrane review and meta-analysis of falls prevention strategies found “uncertain evidence of the effects of bed and chair sensor alarms in hospitals” [15]. The review found no significant reduction in risk of patients becoming “fallers” while in hospital [Relative Risk = 0.93, 95% CI 0.38 to 2.24] based on two trials. There were three relevant trials excluded by the Cochrane review. One, excluded due to the primary outcome being falls by the bedside instead of total falls, found no effect of alarms on fall outcomes by the bedside. Two others, excluded due to alarms being one part of a broader, targeted, multifactorial intervention program, both reported results of no effect [16, 17]. There has also been one trial published subsequent to the Cochrane review which examined the impact of bed alarms triggered by wireless gyroscope and accelerometer technology [18]. It reported that the proportion of patients who became fallers was increased but not significantly during the intervention period of their stepped-wedge trial [odds ratio = 1.54, 95% CI (0.91, 2.61); p = 0.11].

There are additional concerns regarding the use of mobilisation alarms beyond potential ineffectiveness. Falls prevention programs that include alarms cost more than standard care without alarms [19]. Half of all alarms have been reported to be false alarms [20]. Nursing staff have been reported to experience anxiety related to the use of alarms, and have to disrupt important tasks at hand to respond to alarms [21], which may lead to detrimental errors in delivery of care [22] and abandonment of tasks [23]. Although not a physical restraint, the alarms have been cautioned as a form of restraint on patients [24], which can also lead to other adverse outcomes [25].

## Involvement of consumers/End users

Our team conducted a workshop with n = 3 consumer representatives and n = 6 representatives from different health services involved in one of our earlier projects that sought to map and estimate the cost of falls prevention activities being used in hospitals [14]. Workshop participants were provided with the results of our mapping and costing study broken down by individual falls prevention strategies. This study identified that use of falls prevention alarms (including the purchase, location and response to alarms) was the falls prevention activity that consumed the fourth highest amount of resource of the different falls prevention activities undertaken at these six health services. At the time, we also provided evidence from what was the most recent Cochrane review [15] and findings from trials in this field known by the investigators to have been published since that review was completed [16, 19].

The workshop participants unanimously identified mobilisation alarms as the leading candidate for disinvestment, and health service representatives agreed to participate in this project. Workshop participants also questioned whether a “reduced” alarm use condition would be better than the current high alarm approach or the alternative of complete removal. It is important to note that the larger of the trials included in the Cochrane meta-analysis still had some alarm use in the control group [26]. A low alarm use approach may still achieve benefits for a smaller number of patients in terms of potential falls prevention, while minimising the other potential negative aspects of high levels of alarm use (e.g. staff alarm fatigue, patient complaints). From this, we designed the present study which aims to test:

1. whether “Reduced” or “Eliminated” mobilisation alarm conditions are non-inferior to “Current” practice on wards with high levels of mobilisation alarm use for the prevention of falls
2. the superiority of these different conditions for the prevention of falls
3. the economic efficiency of these conditions for the prevention of falls.

## Methods

This trial has been prospectively registered with the Australian New Zealand Clinical Trials Registry: ACTRN12621000823875p. This trial has received approval from the Monash Health Human Research Ethics Committee (number RES-21-0000-468A –approved 27^th^ October 2021).

Any protocol amendments will be submitted to this registry and ethics committee.

### Design

**Fig 4:**
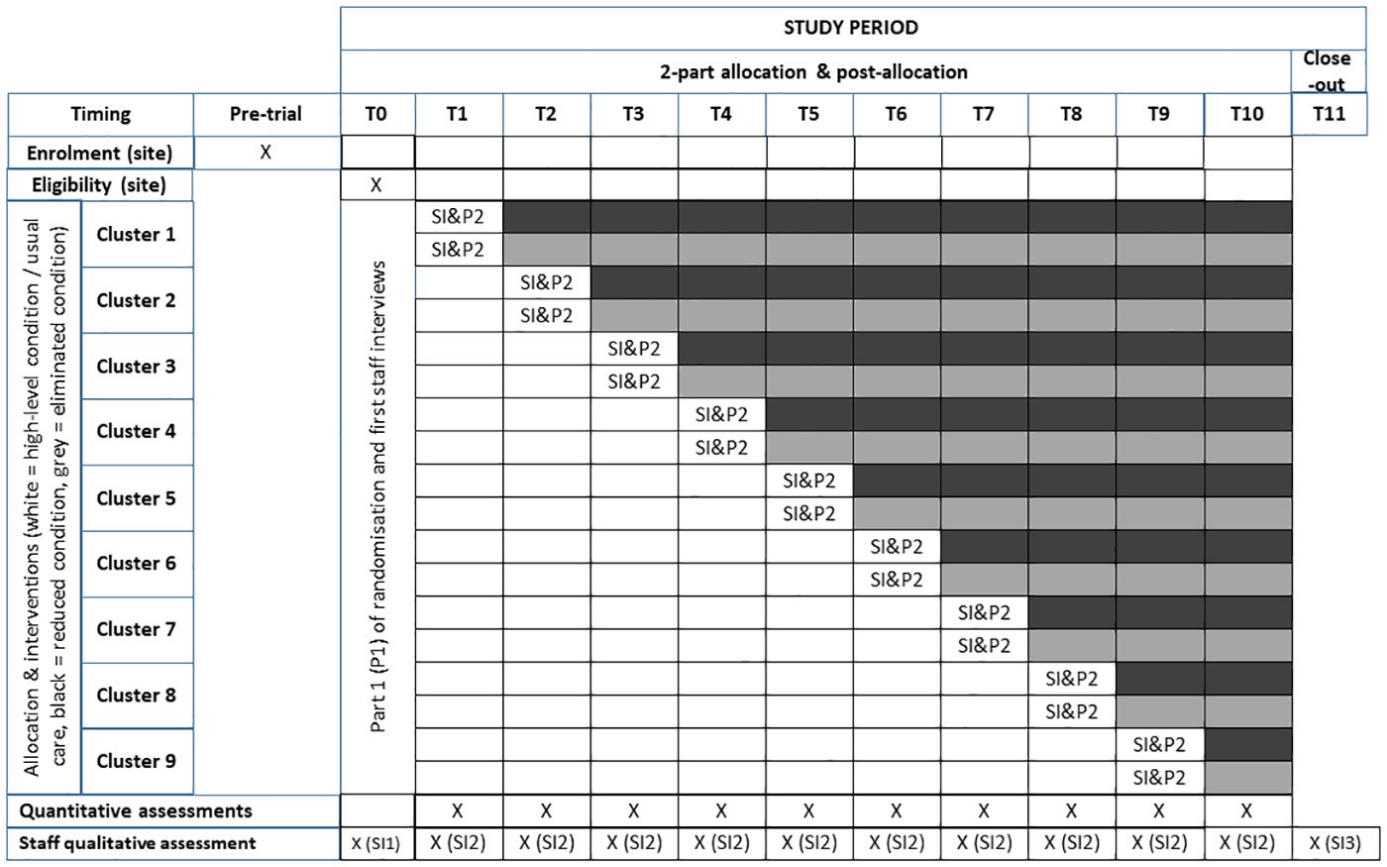
Sprit schedule and trial design with 2-part randomisation (P1&P2) and staff interviews (SI).

This will be a three-group, concurrent, disinvestment, stepped-wedge with an embedded parallel, matched-cluster, randomised trial design. There will be 10 steps in total in the stepped-wedge element of this design (Fig 4 –SPIRIT Schedule).

### Participants & setting

We will recruit 18 adult hospital wards across multiple public or private health services in the State of Victoria in Australia. Inclusion criterion for wards will be a minimum 3% of beds with a mobilisation alarm in place, identified through a daily cross-sectional audit over a two-week period. Emergency, paediatric, mental health, and palliative care wards will be excluded, as will wards with <20 beds. Wards will be paired by type (acute or subacute) to form clusters for initial randomisation. A waiver of requirement to gain patient-level consent for the routinely collected outcomes has been obtained.

### Intervention and control conditions

There will be three conditions:

1. “Current”: Use of alarms will remain unchanged (target: ≥3% of patient bed-days audited involve use of mobilisation alarms)
2. “Reduced”: Use of alarms will be reduced (target: <3% but >0% of patient bed-days audited involve use of mobilisation alarms)
3. ”Eliminated”: Use of alarms will be eliminated (target: 0% of patient bed-days audited involve use of mobilisation alarms)

There will be two combinations of the three conditions for individual participating wards: Current condition followed by Reduced condition, or Current condition followed by Eliminated condition (Fig 4). This trial will be completed within a 12-month time period. Where possible, all mobilisation alarms will be physically removed from wards allocated to the Eliminated condition at the time point of their transition to this condition. Wards allocated to the Reduced condition will be able to retain one or more mobilisation alarms depending on the number of beds on the ward. Routine monitoring of mobilisation alarm use will be undertaken by study data collectors.

#### Primary outcome

Rate of falls by hospital inpatients. We will collect data on both inpatient falls on each participating ward during each calendar month (numerator) and patient-time spent on each participating ward during each calendar month (denominator).

#### Secondary outcomes

*Clinical outcomes*. Rate of fall related injuries as defined by any physical harm resulting from a fall, including bruising, abrasions, lacerations, and fractures. Patient satisfaction as measured by the single item “Overall, how would you rate the care you received while in hospital?” from the Victorian Healthcare Experience Survey [27]. Response categories for this item are Very Good, Good, Average, Poor, Very Poor. Patient sleep quality captured using the Pittsburgh Sleep Quality Index [28]. These latter measures will be included as mobilisation alarms have been found to be a source of patient dissatisfaction [29] and alarm noise is a cause of sleep interruption on hospital wards [30].

*Non-fall related adverse events*. Rate of newly developed (since admission to ward) patient pressure injuries. Rate of medication error. These measures are included as mobilisation alarms have potential to increase patient time spent in bed, and have potential to distract staff while performing other activities that require unbroken concentration respectively. Rates of hospital readmission within 30 days.

*Process outcome—intervention fidelity*. Proportion of patients with mobilisation alarms.

*Process outcome—intervention contamination*. Proportion of patients with other falls-prevention interventions. Interventions to be audited include: falls risk signage inside room, bedside commode, call button within reach, bed sitter, ambulatory aid accessible from bed, regular bed in low position, specialty low-position bed, locked bed, anti-slip floor mat, physical restraint, non-slips socks and non-skid footwear.

*Process outcome—intervention acceptability*. Staff reactions to being involved in this research, immediate concerns regarding the transition to the “Reduced” or “Eliminated” conditions, experiences of the transition, and subsequent reflections of the experience will be collected through qualitative approaches consistent with similar previous disinvestment research [6].

*Economic outcomes*. The economic evaluation will take a limited health-care sector perspective [31] with cost data presented in $AUD during the final year in which the trial is conducted. Clinical costing data and case-mix based payment data (where available) for patients being treated on participating wards during the study period. Costings will be restricted to costs associated with staying on the participating ward. Upstream costs prior to ward admission (for example, days spent in ICU) and downstream costs subsequent to ward discharge (for example, community rehabilitation costs) will not be collected due to these costs being incurred prior to inclusion in the study dataset and due to feasibility issues with collecting data from different organisations not involved in the research. Costs related to the treatment of falls within the health service (for example, if a fall results in a fracture requiring surgery) will be captured from hospital records. Where these data are not available (for example, if a patient is sent to a different hospital for surgery), then the mean casemix payment for the procedure anticipated to be undertaken will be imputed, based on information available in the patient record or incident report describing the injury. A determination of the anticipated procedure will be made by two team members blinded to the condition under which the injury occurred.

### Procedures

#### Recruitment/Screening

Purposive sampling will be used to recruit health services into this trial on the basis of participation in our earlier research mapping of falls intervention use, and participation in our consumer / stakeholder representative workshop where mobilisation alarms were selected as the leading candidate for disinvestment. These health services include four large public health services and two private health services that combined account for nearly 10% of inpatient health care service provision in Australia. Each potential ward identified for potential inclusion will be screened for eligibility over a 2-week period with daily cross-sectional audits of mobilisation alarm use; results will be averaged to determine if the ward meets the ≥3% threshold. Wards will be recruited from hospitals in multiples of 2.

#### Random allocation

A two-stage randomisation process will be employed. In the first stage, ward pairs will be formed based on type (acute vs subacute). Ward pairs form a cluster that will be allocated to starting positions within the stepped-wedge design by an investigator blinded to ward identity (each ward will be provided with a pseudonym known by a separate investigator to mask identity) using a computer-generated random number sequence. The identity of each ward will then be revealed and participating wards will be notified of their duration of exposure to the “Current” condition. There will be nine pairs of wards (18 wards in total) involved in the trial. The stepped-wedge component will have 10 calendar-month time periods, with one cluster / ward pair transitioning from the “Current” condition each calendar month.

The second stage of the randomisation involves splitting the ward pairs to promote comparability in fall rates between “Reduced” and “Eliminated” conditions for the embedded, parallel, cluster randomised trial component of the study. We will adapt Frane’s dynamic randomisation approach [32] to minimise the difference between wards in the rate of falls on wards during their earlier exposure to the “Current” condition such that wards allocated to the “Reduced” and “Eliminated” conditions are collectively as comparable as possible on the primary outcome to this point. The first three ward pairs will be split between “Reduced” and “Eliminated” conditions using simple, computer generated randomisation undertaken by an investigator blinded to ward identity. After this, ward pairs transitioning out from the “Current” condition will be temporarily split in both possible ways, and a mixed-methods analysis using all available falls rate data from the “Current” condition period will be undertaken with allocation status (“Reduced” or “Eliminated”) as the independent variable of interest. The allocation approach that has the highest p-value from the two analyses will then be adopted. This will be repeated for each step of the design.

#### Setting stopping rules, non-inferiority margins, and data monitoring

Conducting a disinvestment trial in the context of uncertainty in intervention ineffectiveness creates risk of harm to patients during the trial period. A project governance committee including at least one key representative from each participating health service will be tasked with identifying stopping rules for this trial. Stopping rules will be tied to 95% confidence intervals of the difference between the “Current” condition and “Reduced” or “Eliminated” condition fall rates exceeding a non-inferiority margin selected by this committee prior to trial commencement. Non-inferiority margin deliberations of the project governance committee will be guided by the principle of identifying the minimum reduction in rate of falls that mobilisation alarms would need to achieve in order to justify the costs (economic, and estimated adverse effects on patient care, e.g. patient dissatisfaction) of their provision at “Current” levels compared to “Reduced” and “Eliminated” levels. A data monitoring committee will review the results of stopping rule checks each month, any adverse events, and the final analysis. The data monitoring committee will be comprised of two investigators and one representative from each of the health services in the study.

#### Clinical staff engagement

Prior to trial commencement, staff members from each participating ward and health service will be provided with information relating to the use of mobilisation alarms for preventing falls. This will include information relating to the efficacy of mobilisation alarms for the prevention of falls taken from the most recent Cochrane review [15] and trials or reviews published subsequently that substantively add to what was already published in the Cochrane review. It will also include information from our mapping / costing study of falls prevention [14] and a summary of other published impacts of mobilisation alarm use [15–17, 19–25]. It will then provide a description of the research approach, and provide an opportunity for staff to raise concerns and discuss appropriate strategies for local communication to staff who may be impacted.

Staff will receive further trial reminders prior to their transition away from the “Current” condition. At trial conclusion, staff will receive a summary of trial findings.

#### Consumer engagement

Prior to trial commencement, n = 18 community-dwelling people over the age of 60 will be recruited via social media to discuss how they feel about hospitals changing their current practice to remove a part of care that has:

1. clear evidence of ineffectiveness
2. clear evidence of inefficiency (i.e. although effective, it is not cost effective)
3. uncertain evidence of effectiveness
4. uncertain evidence of effectiveness, but that during the removal from care, evidence is generated as to its effectiveness

In each case, participants will be asked about how they feel patients and their family / care givers should be informed of this and potentially involved in the original decision-making process. This progression of logic was chosen to help promote consumer participant understanding of the nuanced framework through which our disinvestment study is being conducted. Participants will then be provided with information about mobilisation alarm use, uncertainty on their effectiveness/ineffectiveness, and potential adverse consequences of their use. They will then be asked the same questions as to how they feel about disinvestment from use of mobilisation alarms in the context of a trial being conducted at the same time to establish their effectiveness / ineffectiveness, and how they feel patients and family / care givers should be informed of this and potentially involved in the decision-making process. Feedback gathered from this investigation will inform consumer communication strategies used in the trial.

#### Outcome measure collection

There will be four outcome collection approaches. Need for consent was waived for approaches 1 and 2. Approaches 3 and 4 require to be provided in written and verbal format by a project research assistant, with written consent without additional witnessing.

1. Extraction from hospital records (patient medical records, incident report databases, administrative databases): Records will be audited on a weekly basis by on-site research assistants at each health service.
2. Direct observation: The rate of use of mobilisation alarms and other falls prevention interventions will be recorded 3 times a week during the period of the trial.
3. Interviews with hospital staff: Nurse unit managers (NUM) will be surveyed once a week for their documentation of falls within that week to supplement hospital records of falls and incidents. Consenting staff on each participating ward will be interviewed at different time points during the trial regarding their attitudes and experiences with the trial (Fig 4).
4. Interviews with a sub-sample of patients: During the trial, study wards will be visited on randomly selected days each week and all patients who are estimated to be within 36-hours of discharge will be approached to complete patient satisfaction and sleep quality questionnaires.

Mapping of each outcome to the data collection approach is presented (Table 1). Primary outcome falls and hospital bed-day data will be collected in line with approaches previously demonstrated to enhance capture of these outcomes [33, 34]. Data will be collected or extracted locally at each site and submitted to the trial coordinator via a online data entry tool for secure cloud-based storage and ongoing processing by investigators for monthly stopping rule checks and final analysis.

**Table 1:** Outcome measures and corresponding data collection approaches.

Outcome	Outcome Type	Data Collection Approach(es)
Rate of Falls	Primary Clinical Outcome	Extraction from hospital records Interview with nurse unit managers (NUM)
Rate of falls-related injuries	Secondary Clinical Outcome	Extraction from hospital records
Patient satisfaction with care–Victorian Patient Satisfaction Survey [27]	Secondary Clinical Outcome	Interview with patient sub-sample
Patient sleep quality–Pittsburgh Sleep Quality Index [28]	Secondary Clinical Outcome	Interview with patient sub-sample
Rate of newly developed pressure injuries (since admission to ward)	Secondary Outcome—Non-falls Related Adverse Events	Extraction from hospital records
Rate of medication error	Secondary Outcome—Non-falls Related Adverse Events	Extraction from hospital records
Rate of hospital readmission within 30 days	Secondary Outcome—Non-falls Related Adverse Events	Extraction from hospital records
Proportion of patients with mobilisation alarms	Secondary Outcome—Intervention Fidelity	Direct observations of ward beds
Rate of use of “other” confounding falls-prevention interventions	Secondary Outcome—Intervention Contamination	Direct observations of ward beds
Casemix payments to hospital	Secondary Intervention Outcomes	Extraction from hospital records
Procedures subsequent to falls-related injury	Secondary Intervention Outcomes	Extraction from hospital records
Staff attitudes to involvement in trial	Other Secondary Outcomes	Interview with NUMs and ward staff at the start of the trial
Staff concerns regarding transition from Current condition to either Reduced or Eliminated conditions	Other Secondary Outcomes	Interview with NUMs and ward staff prior to transition
Staff experiences of transition and intervention	Other Secondary Outcomes	Interviews with NUMs and ward staff at 1-month post-trial follow-up

#### Blinding

Personnel collecting data and ward staff who will be interviewed will not be blinded to the condition status of participating wards during the trial. Personnel involved in randomisation will be blinded to the identity of wards being randomised. Investigators involved in the statistical analysis will be blinded to the allocation status of the wards involved through use of multiple mock condition and time-period codes included in the analysis dataset and the analyst being requested to analyse each combination (including mock and real) and forming data interpretations based on each.

### Data analysis

The rate of falls will be compared between conditions (“Current” vs “Reduced” vs “Eliminated”) based on data from the entire trial at the summative ward-month level (i.e. one unit of measure per ward per month) using mixed-effects, general linear model analyses. Ward and cluster (pair) will be entered as random effects, with calendar month entered as a categorical fixed effect consistent with recommendations for analysis of stepped-wedge trials [11]. Primary outcome data (rate of falls) are planned to be treated as continuous, normally distributed data when considered at the ward-month level. Classifications of inferiority / non-inferiority / uncertainty of inferiority will be made relative to one-tailed, 95% confidence, non-inferiority margins developed during this research. All pair-wise between-condition contrasts will be examined for superiority using two-tailed 95% confidence intervals (investigation of non-inferiority does not preclude investigation of superiority and can be done so without statistical penalty [35]). Visual investigation of falls data will be undertaken using “transition-relative” line graphs of both raw data and residuals post analysis of calendar month [36, 37]. If indicated by these analyses, data transformations or inclusion of interaction terms with time as a continuous variable (i.e. to detect intervention effects that change over time since transition) will be investigated but classified as secondary analyses. Secondary outcomes will be similarly compared between conditions using mixed-effects, general linear model analyses with the exception of data that are collected from the sub-sample of patients as these data will be analysed at the patient level rather than the summative ward-month level. Intention-to-treat analyses will be pursued with multiple imputation for any missing data for the primary outcome if it arises.

#### Qualitative analyses

The Framework Method will be used to systematically organise and categorise qualitative data captured examining staff experiences of being involved in this disinvestment project and how their perspectives on involvement in this project change over time [38, 39]. Final themed categories will be cross-checked with representatives of health services within our project governance structure and with research team members for authenticity.

#### Economic analyses

An incremental cost effectiveness analysis examining the cost per fall prevented per 1000 patient-days of observation time will be undertaken across the three pairwise comparisons possible. It will be a trial-based approach using health-service resource use (casemix funding payment data) and falls rate data from the present study in the base case. It will integrate economic data collected from previous cost-of-falls investigations [40], and studies that have measured the cost of delivering falls prevention interventions including mobilisation alarms and other confounding falls interventions [14, 37]. Mean difference in health care resource use between conditions will be estimated using linear mixed model analyses [41]. Each pair-wise incremental cost effectiveness ratio (ICER) will be presented as a point estimate with confidence ellipses on a cost-effectiveness plane to visually demonstrate if there is a dominant condition of the three examined. Confidence intervals around the individual ICERs will be calculated using the bootstrap method (5,000 repetitions) [42]. The ICER will use the central limit theorem to generate confidence ellipses and cost effectiveness acceptability curves [42] to inform the probability that disinvestment in mobilisation alarms is less costly and more beneficial compared with usual care alone. One-way sensitivity analyses will be pursued including use of clinical costing data instead of casemix payment data to measure health service resource use, varying the estimated cost of confounding falls interventions, and by varying the cost-per fall (based on whether falls are assumed to increase length of stay or not [40]).

### Sample size considerations

This study will involve 18 hospital wards, each observed over 10 ward-month (calendar month) time periods. The unique attributes of this concurrent, multi-group stepped-wedge design led us to undertake bootstrap power analysis simulations using Stata MP version 15.0 [43] to examine the likely sufficiency of the sample size in this design to address the research hypotheses related to our primary outcome. We assumed a mean (sd) baseline (“Current” condition) rate of falls per ward-month period of 8 (4) and an ICC of 0.6 based on previous research [12, 16, 17]. We randomly generated data meeting these requirements for 1000 stimulated replications of this design. We then randomly sampled with replacement individual studies from this pool 1000 times. We varied the possible impact of the “Reduced” and “Eliminated” conditions in these simulations to estimate the detectable difference to achieve 80% of simulations rejecting the null hypothesis at a 95% confidence level in the planned one-tailed non-inferiority analyses and two-tailed superiority analyses.

For non-inferiority analyses involving the “Current” condition: A non-inferiority margin of 1.8 falls per ward-month led to classification of the “Reduced” or “Eliminated” conditions as being non-inferior to the “Current” condition in 80% of simulations (i.e. 80% power) where the simulation was designed to have no mean difference between groups.

For superiority analyses involving the “Current” condition: A simulated mean difference of 2.1 falls per ward-month achieved superiority (rejection of the null) in 80% of simulations.

For non-inferiority analysis of “Reduced” vs “Eliminated” conditions: A non-inferiority margin of 1.9 falls per ward-month led to classification of the “Eliminated” conditions as being non-inferior to the “Reduced” condition in 81% of simulations (i.e. 81% power) where the simulation was designed to have no mean difference between groups.

For superiority analysis of “Reduced” vs “Eliminated” conditions: A simulated mean difference of 2.4 falls per ward-month achieved superiority (rejection of the null) in 80% of simulations.

## Discussion

Both disinvestment and the stepped-wedge design are relative newcomers in the fields of medical and policy research [10]. The use of disinvestment-specific, stepped-wedge trial designs has recently been recorded in the falls prevention field. The advance presented in this paper has been in their application to situations where multiple intervention / control conditions are of interest using a concurrent, stepped-wedge approach. The choice of mobilisation alarm as a research target was informed by the uncertain evidence of effectiveness [15], evidence of high false alarm rates [20], and high cost for their use in hospitals [14]. The inclusion of an alternative intervention condition, reducing alarm use rather than only eliminating it, was driven by health service stakeholder and consumer input and the absence of an eliminated condition in previous research, making it an ideal candidate for us with this research design.

Limitations with use of this design have become apparent since the COVID-19 outbreak. Disinvestment can be a particularly challenging research paradigm for health service staff members [5, 6], while stepped-wedge designs are vulnerable to the impact of external events and large-scale changes in health services. COVID-19 has led to staff infections / anxiety and changes to the patient casemix in wards that were to have been a part of this trial. Consequently, our team of investigators has postponed commencement of this trial until the impact of the COVID-19 pandemic on our participating health services has been brought under control.

The simulation-based power analyses undertaken for our application area indicated similar but slightly greater statistical power for comparisons made through the stepped-wedge portion of the design as compared to comparisons through the embedded, parallel, cluster randomised trial portion of the design. In each case, the superiority analyses will likely generate 95% confidence intervals substantially narrower than previous, large scale, parallel, cluster randomised controlled trials involving similar numbers of hospital wards and time scales, as the power to detect differences were generally less than a 25% change in fall rates [16, 17]. Further research would be needed to investigate if this relationship holds for other applications, particularly where there may be variations in the intra-cluster correlation coefficient values employed.

## Supporting information

S1 File (DOCX)

S1 Checklist SPIRIT 2013 Checklist: Recommended items to address in a clinical trial protocol and related documents. (DOC)
